# Supplementary material for: Biomimetic mercury immobilization by selenium functionalized polyphenylene sulfide fabric
Source: Nat Commun. 2024 Feb 12;15:1292. doi: 10.1038/s41467-024-45486-7 (PMC10861514; doi:10.1038/s41467-024-45486-7)
Supplement: Supplementary file 1 — Supplementary Information [file 41467_2024_45486_MOESM1_ESM.pdf]

Supplementary Information

Biomimetic Mercury Immobilization by Selenium Functionalized Polyphenylene Sulfide Fabric

Hailong Li[1], Fanyue Meng[1], Penglin Zhu[1], Hongxiao Zu[1], Zequn Yang[1], Wenqi Qu[1], Jianping Yang[1]*

1. School of Energy Science and Engineering, Central South University, Changsha, 410083, China

*Corresponding author.

TEL:+86-13203136046

Fax: +86-731-88879863

Email: jpyang@csu.edu.cn

List of Supplementary Tables

Supplementary Table 1. **Experimental conditions.**

Supplementary Table 2. **Hg^0 adsorption capacities of different adsorbents.**

Supplementary Table 1. Experimental conditions

Experiments	Sorbents	Gas components	Temperature (°C)	GVSH (m min^{-1})	Hg^0 concentration (μg m^{-3})
Set I	PPS, Se/PPS-P, Se/PPS-I, SeO_3^{2}-PPS, Black Se powder, Red Se powder	N_2	125	5.66	1000
Set II	Se/PPS-I (0%, 0.1%, 0.25%, 0.5%, 1%NaOH concentration; 2.75%, 5.5%, 11%, 22%, 44% SeO_3^{2-} concentration)	N_2	125	5.66	1000
Set III	PPS, Se/PPS-I	N_2	25, 50, 75, 100, 125, 150	5.66	1000
Set IV	Se/PPS-I	N_2<br>80%N_2+20%O_2<br>N_2+8%/14%/20% H_2O<br>N_2+2000ppm/1%/2%SO_2<br>N_2+5%O_2+8H_2O +2000ppmSO_2	125	5.66	1000
Set V	Se/PPS-I	N_2	125	5.66	1000

	(with and without covering by particulate matter)				
Set VI	Se/PPS-I	N_2	125	0.57, 2.83, 5.66, 11.32	1000
Set VII	Se/PPS-I	N_2	125	1.13, 5.66	1000
Set VIII	Se/PPS-I<br>Polyester<br>Se/Polyester	N_2	125	5.66	1000

Supplementary Table 2. Hg^0 adsorption capacities of different adsorbents.

Adsorbents	Carrier gas	Breakthrough threshold/Test time (h)	Hg^0 adsorption capacities (mg g^{-1})	Hg^0 concentration	References
Se/PPS	N_2	80%/96h	1203.4	1000	This work
CuSe	N_2	80%/13h	80.2	1000	1
CuSe/CAU-10-U	N_2	70%/100h	302.23	100	2
Se/ZIF-8	N_2	100%/NA	40.8	200	3
CuSe/ZIF-8	N_2	100%/50h	315.2	200	3
WSe_2	NA	NA/80h	30.6	560	4
CuSe/g-C_3N_4	NA	80%/11h	9.1	180	5
Se/MIL-101	N_2	80%/220h	148.19	1000	6
$Fe_3O_{4-X}Se_y$	SFG1	100%/50h	8.8	65	7
CuS	N_2	80%/5h	103.73	1000	8
Fe/ZnS	Air	47%/60h	8.65	1600	9
$Co_xZn_{1-x}S$	N_2+O_2	50%/NA	46.01	1100	10
Co_3S_4	N_2	50%/24h	43.03	1300	11
Fly ash	NA	100%/3h	0.0103	10	12
S-AC	SFG2	49%/16.25 h	1.156	10	13
Cl-AC	Air	NA/8h	0.879	35	14
Br-AC	N_2	100%/2.5h	1.53	NA	15
SBA-15-Ag	NA	NA	0.06	77.6	16
Ag-beads	N_2	1%/NA	0.0002	447	17

Au-beads	N_2	1%/NA	0.0029	447	17
Ag/Graphene	N_2	NA	4.2	500	18
MnO_2@MOS	N_2+O_2	20%/32h	1.52	300	19
$CeO_2/MnO_x/TiO_2$	SFG3	NA	9.4	30-50	20
CeO2	N_2	NA/2h	0.1	80	21
$CeO_2/ZnIn_2S_4$	N_2+O_2	NA/10h	0.9	80	21

SFG1: 4%O_2, 500 ppm SO_2 and 8% H_2O

SFG2:14% CO_2, 6% O_2, 10% H_2O, 50 ppm HCl, 200 ppm SO_2 and 200 ppm NO

SFG3: 400 ppm NO and 400 ppm CO

List of Supplementary Figures

Supplementary Fig. 1. Characteristic of pristine PPS. (a) photo and (b) SEM image of pristine PPS.

Supplementary Fig. 2. Distribution of Selenium on Se/PPS prepared with different methods. EDS mapping for the (a) Se/PPS-P and (b) Se/PPS-I.

Supplementary Fig. 3. The photo of Se/PPS-I with a size of 100 cm × 200 cm.

Supplementary Fig. 4. Structure for quantum chemical calculations. The structure of (a) PPS monomer, (b) SeO_3^{2-} anion, and (c) Se molecule (the atomic coordinates of the optimized computational models refer to Supplementary Data).

Supplementary Fig. 5. The water contact angle of PPS.

Supplementary Fig. 6. SEM image of selenium powder.

Supplementary Fig. 7. EDS mapping images of SeO_3^{2-}/PPS.

Supplementary Fig. 8. Selenium loading amount of Se/PPS-I measured by ICP-MS. Effect of (a) NaOH and (b) SeO_3^{2-} concentration on the selenium loading amount on Se/PPS-I measured by ICP-MS.

Supplementary Fig. 9. Variation of solution colors during the *in-situ* synthesis process with adding different NaOH dosage (relative to SeO_3^{2-} concentration). (a) selenium precursor solution before adding NaOH, (b) solution after adding NaOH (elemental selenium has been formed), (c) adding PPS for loading elemental selenium, (d) residual solution after taking out PPS, (e) the as-prepared Se/PPS-I samples.

Supplementary Fig. 10. Thermal stability of different samples. TG-DTA curve for (a) PPS, (b) Se powder.

Supplementary Fig. 11. Stability of selenium on the Se/PPS. (a) The weight change of Se/PPS purging with N_2 for 14 days, (b) The selenium content of Se/PPS before and after purging with N_2 for 14 days

(determined by inductively coupled plasma mass spectrometry and all samples were determined three times).

Supplementary Fig. 12. EDS mapping of Se/PPS-I.

Supplementary Fig. 13. Effect of PPS decomposition on the investigation of mercury species. (a-d) TPD spectrum of mercury species over HgSe and HgS compounds with and without PPS, (e) Stability of mercury analyzer with and without PPS decomposition in the carrier gas.

Supplementary Fig. 14. Hg^0 adsorption performance of SeO_3^{2-}/PPS and pristine PPS.

Supplementary Fig. 15. Hg^0 adsorption performance of Se/PPS-I prepared with different NaOH dosages at 75 °C. The selenium content has been normalized by adjusting the adsorbent dosage.

Supplementary Fig. 16. Hg^0 adsorption performances of powdery red and black selenium.

Supplementary Fig. 17. Hg^0 adsorption performances of different samples at varied temperatures. (a) PPS, (b) Se/PPS-I, (c) Polyester, (d) Se/Polyester.

Supplementary Fig. 18. Kinetics models for Hg^0 adsorption over Se/PPS-I. (a) Pseudo-first-order and (b) Pseudo-second-order kinetic models, (c) Intra-particle diffusion model, and (d) Elovich model.

Supplementary Fig. 19. Hg^0 adsorption performance of Se/PPS under different atmospheres (a) different H_2O concentration, (b) different SO_2 concentration, (c) H_2O and SO_2 co-existed

Supplementary Fig. 20. A proposed scheme for Hg^0 removal and recovery by Se/PPS-I.

Supplementary Fig. 21. Hg^0 removal performances of Se/PPS with and without covering by particulate matter. (a) Se/PPS covered by fly ash, (b) Hg^0 removal performances of Se/PPS with and without covering by fly ash.

Supplementary Fig. 22. The Hg^0 adsorption capacity of Se/PPS as a function of time.

Supplementary Fig. 23. Schematic diagram of mercury and selenium recovery system.

Supplementary Fig. 24. Photo of mercury and selenium recovered from spent Se/PPS-I. (a) liquid mercury, (b) selenium.

Supplementary Fig. 25. XPS spectra over fresh and Hg-laden Se/polyester. (a) Se 3d, (b) Hg 4f of XPS spectra.

Supplementary Fig. 1. Characteristic of pristine PPS. (a) photo and (b) SEM image of pristine PPS.

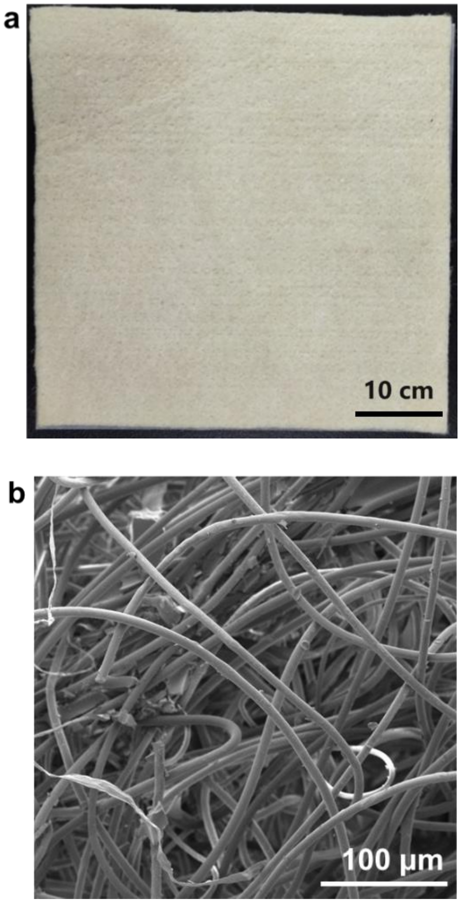

Supplementary Fig. 2. Distribution of Selenium on Se/PPS prepared with different methods. EDS mapping for the (a) Se/PPS-P and (b) Se/PPS-I.

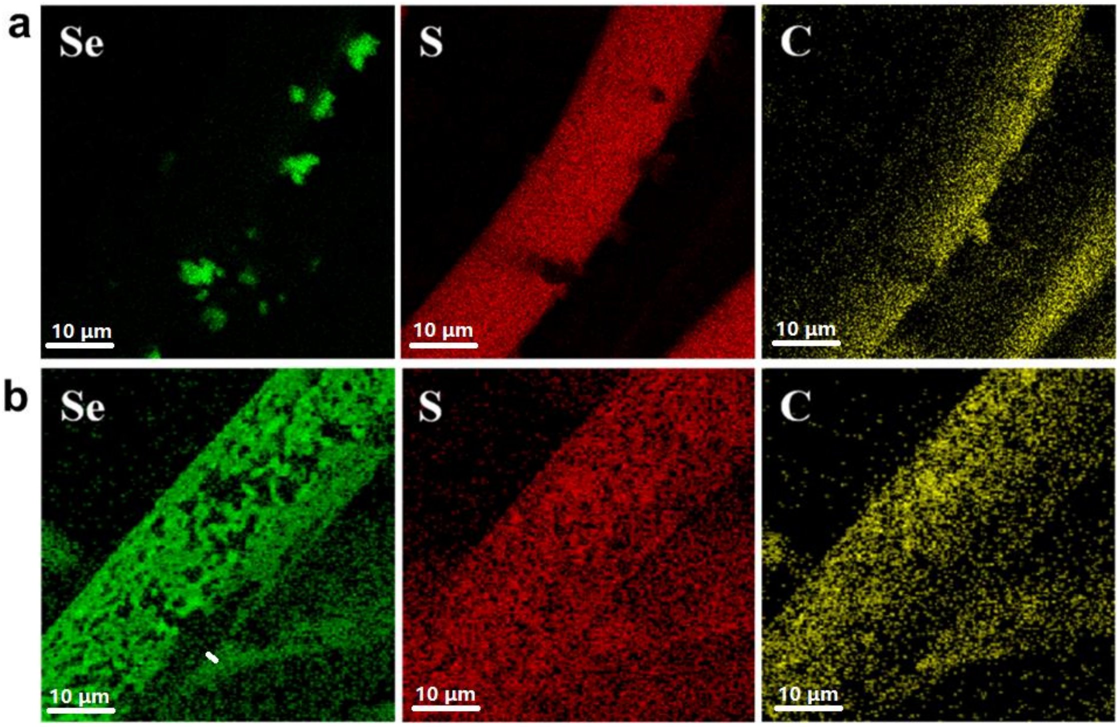

Supplementary Fig. 3. The photo of Se/PPS-I with a size of 100 cm × 200 cm.

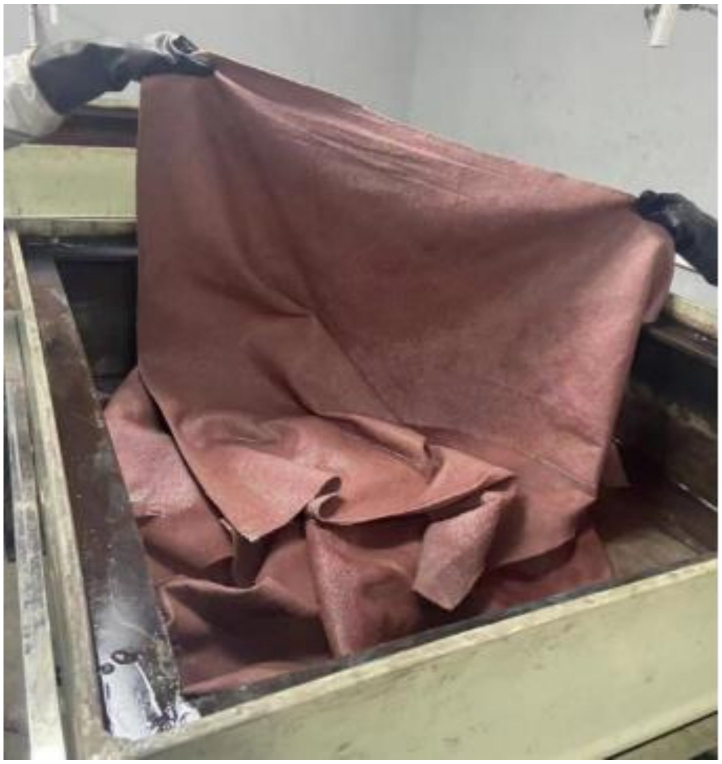

Supplementary Fig. 4. Structure for quantum chemical calculations. The structure of (a) PPS monomer, (b) SeO_3^{2-} anion, and (c) Se molecule (the atomic coordinates of the optimized computational models refer to Supplementary Data).

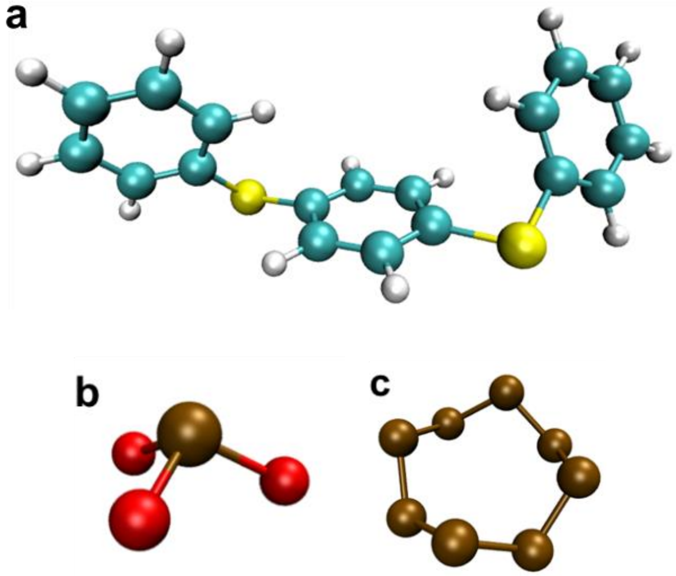

Supplementary Fig. 5. The water contact angle of PPS.

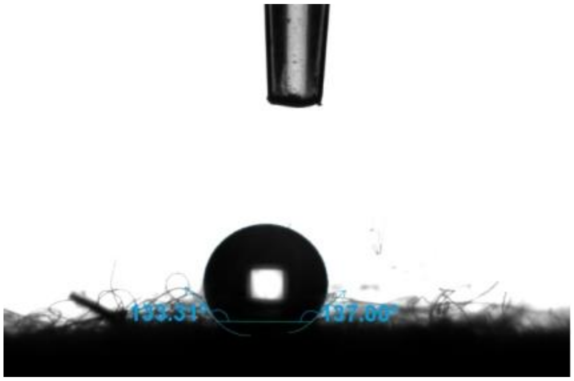

Supplementary Fig. 6. SEM image of selenium powder.

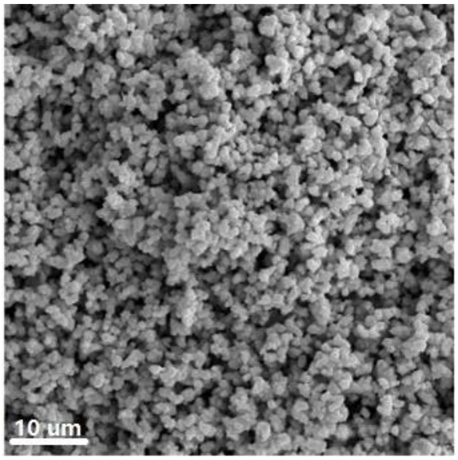

Supplementary Fig. 7. EDS mapping images of SeO_3^{2-}/PPS.

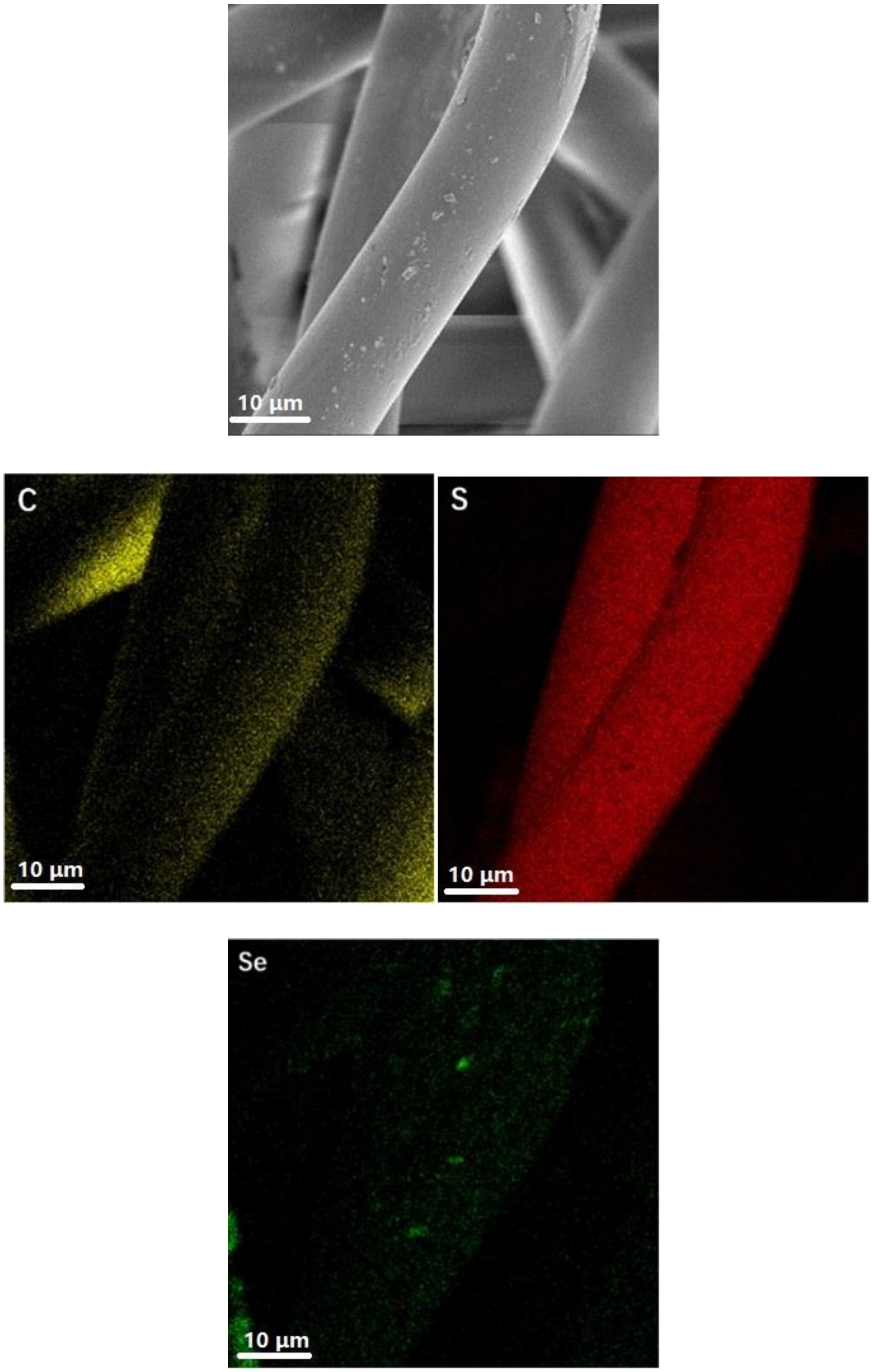

Supplementary Fig. 8. Selenium loading amount of Se/PPS-I measured by ICP-MS. Effect of (a) NaOH and (b) SeO_3^{2-} concentration on the selenium loading amount on Se/PPS-I measured by ICP-MS.

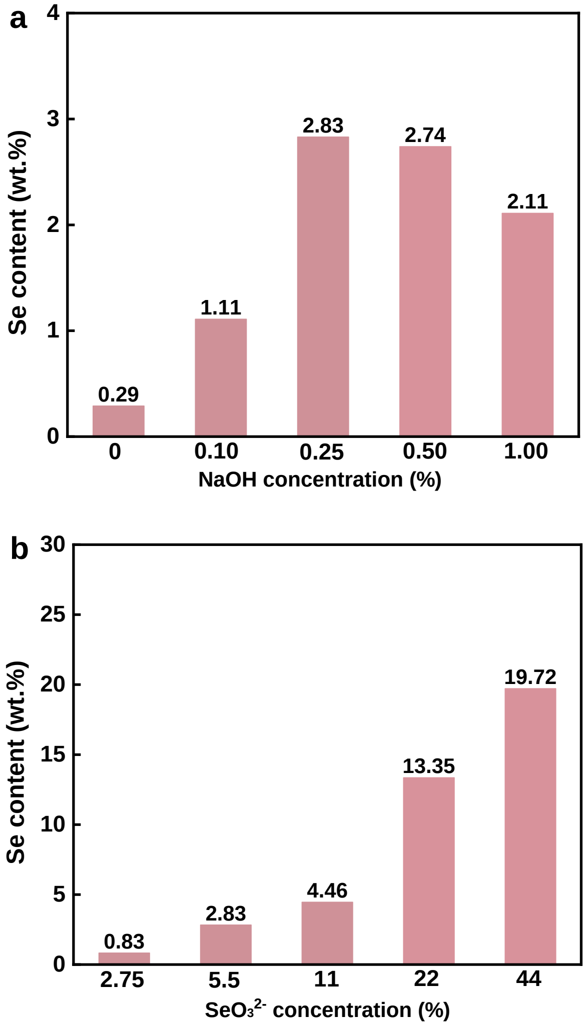

Supplementary Fig. 9. Variation of solution colors during the *in-situ* synthesis process with adding different NaOH dosage (relative to SeO_3^{2-} concentration). (a) selenium precursor solution before adding NaOH, (b) solution after adding NaOH (elemental selenium has been formed), (c) adding PPS for loading elemental selenium, (d) residual solution after taking out PPS, (e) the as-prepared Se/PPS-I samples.

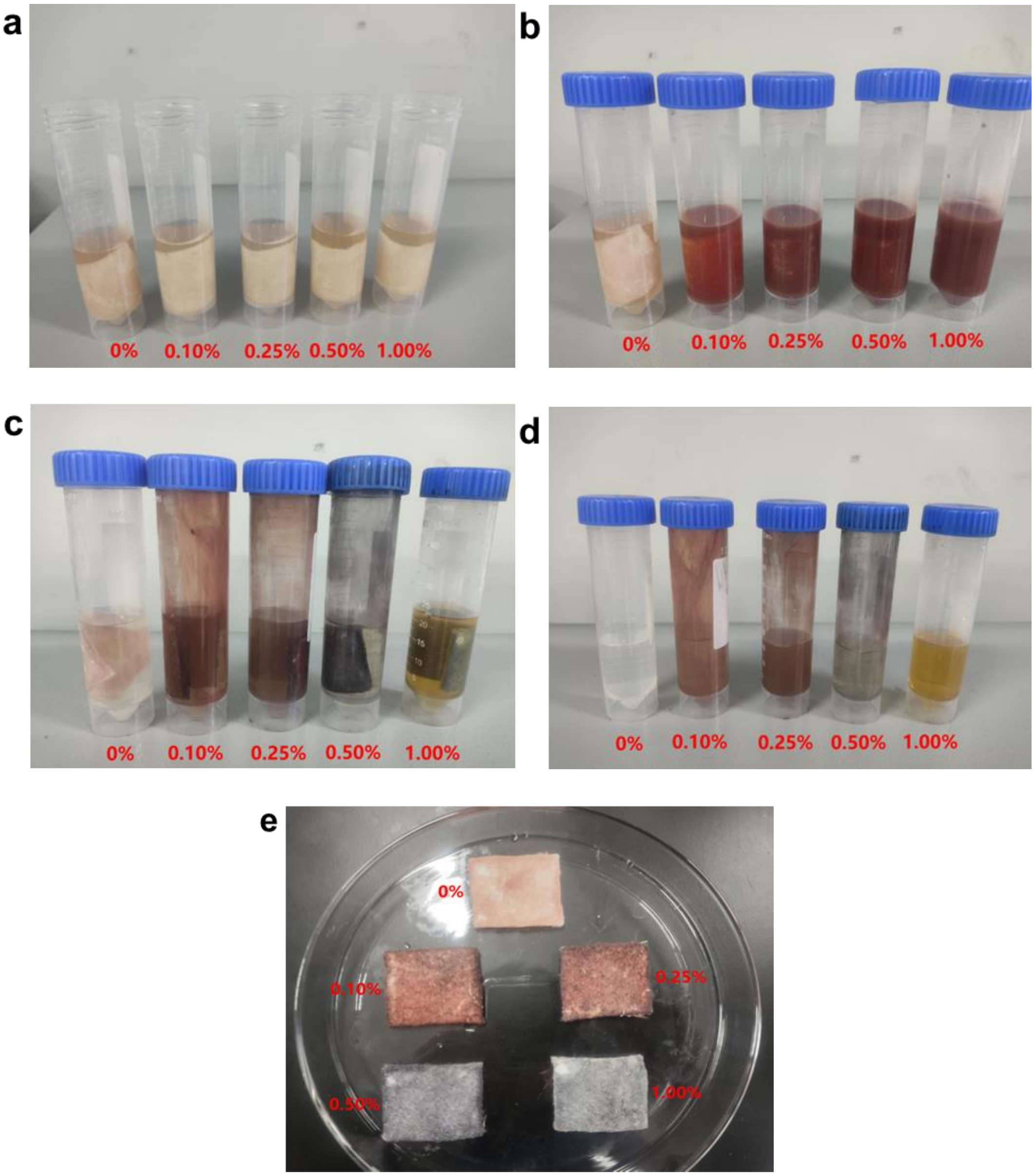

Supplementary Fig. 10. Thermal stability of different samples. TG-DTA curve for (a) PPS, (b) Se powder.

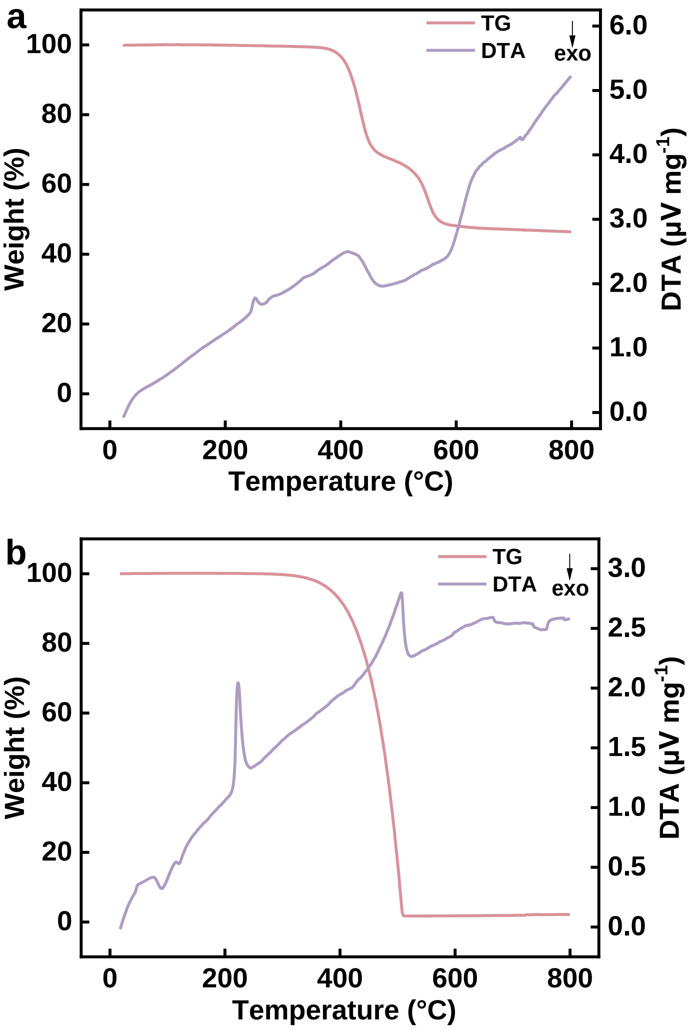

Supplementary Fig. 11. Stability of selenium on the Se/PPS. (a) The weight change of Se/PPS purging with N_2 for 14 days, (b) The selenium content of Se/PPS before and after purging with N_2 for 14 days (determined by inductively coupled plasma mass spectrometry and all samples were determined three times).

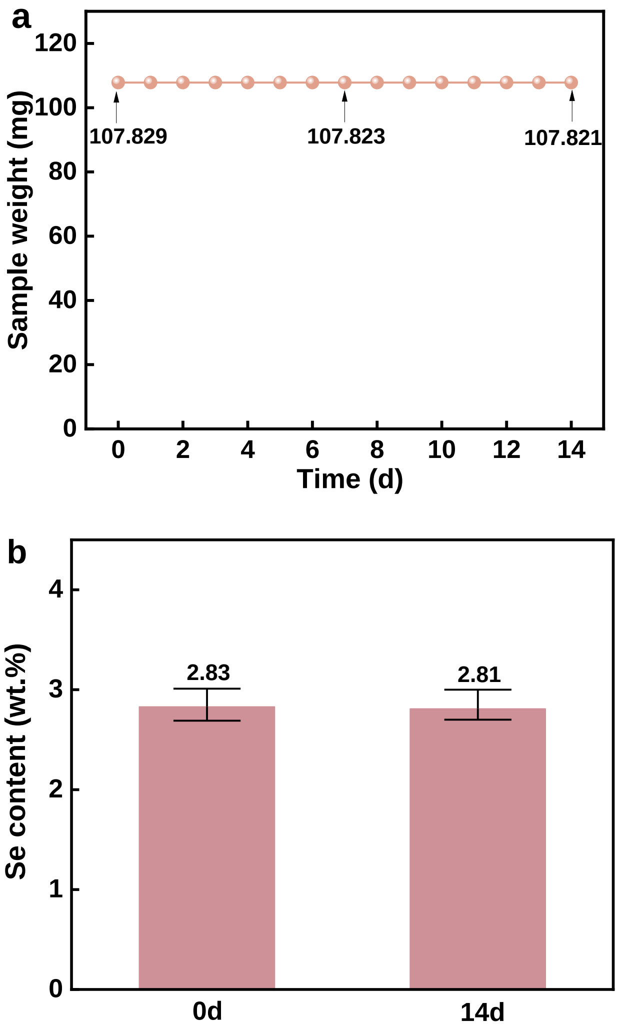

Supplementary Fig. 12. EDS mapping of Se/PPS-I.

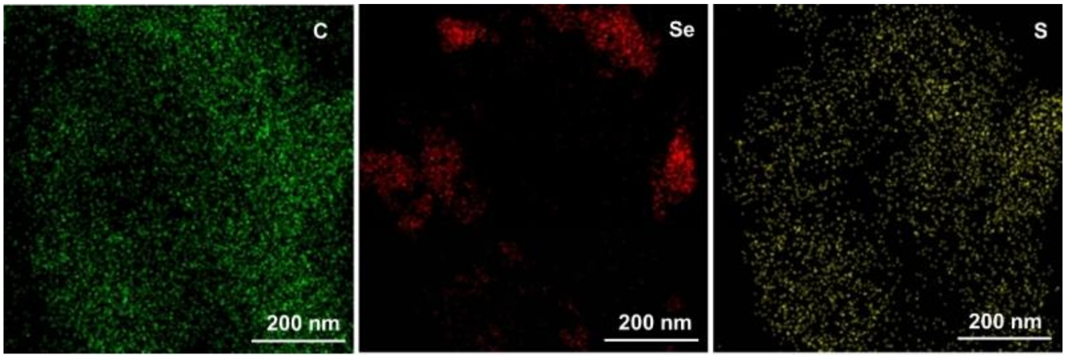

Supplementary Fig. 13. Effect of PPS decomposition on the investigation of mercury species. (a-d) TPD spectrum of mercury species over HgSe and HgS compounds with and without PPS, (e) Stability of mercury analyzer with PPS decomposition components in the carrier gas.

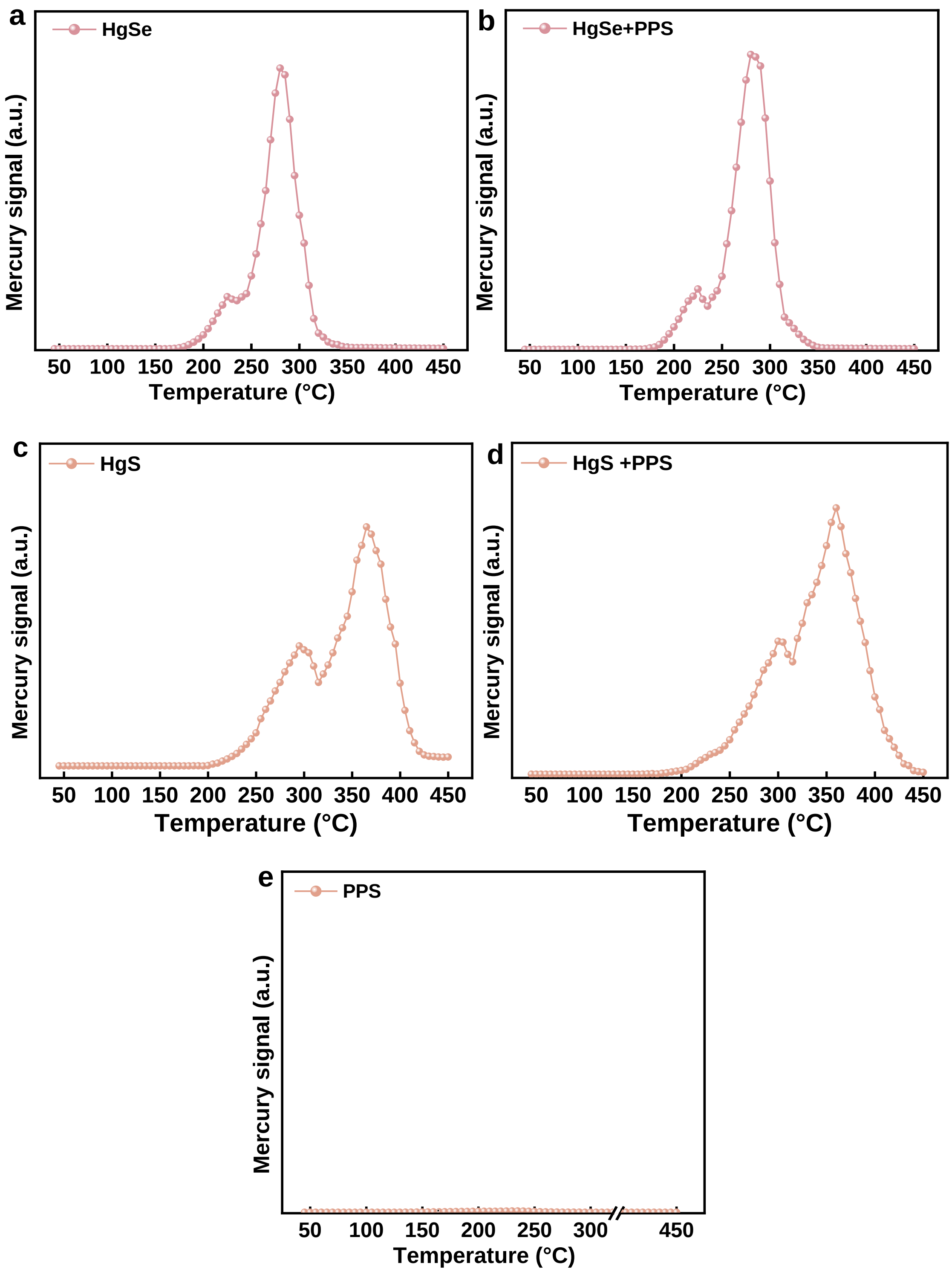

Supplementary Fig. 14. Hg^0 adsorption performance of SeO_3^{2-}/PPS and pristine PPS.

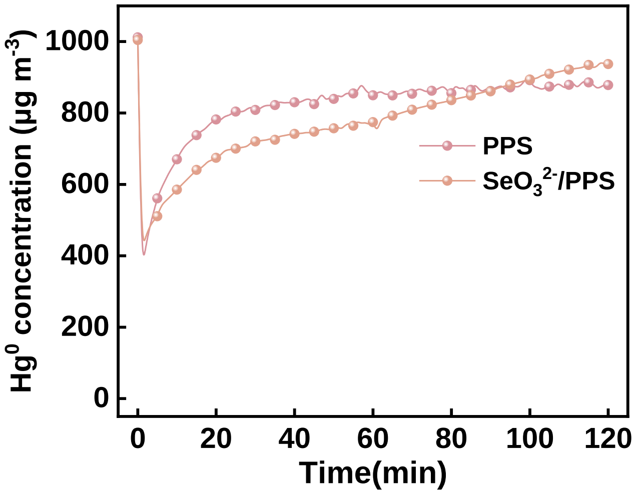

Supplementary Fig. 15. Hg^0 adsorption performance of Se/PPS-I prepared with different NaOH dosages at 75 °C. The selenium content has been normalized by adjusting the adsorbent dosage.

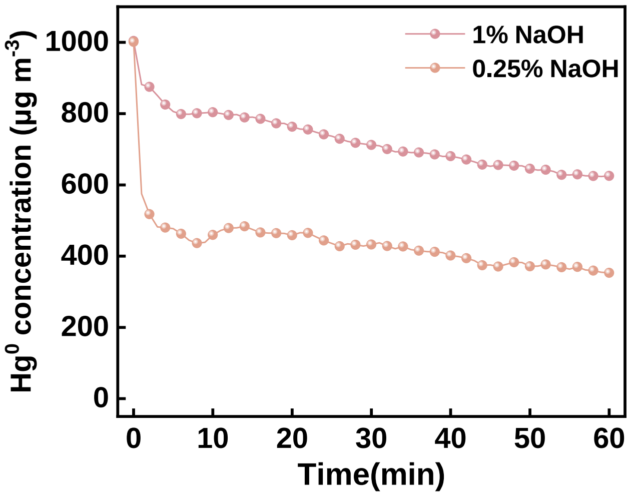

Supplementary Fig. 16. Hg^0 adsorption performances of powdery red and black selenium.

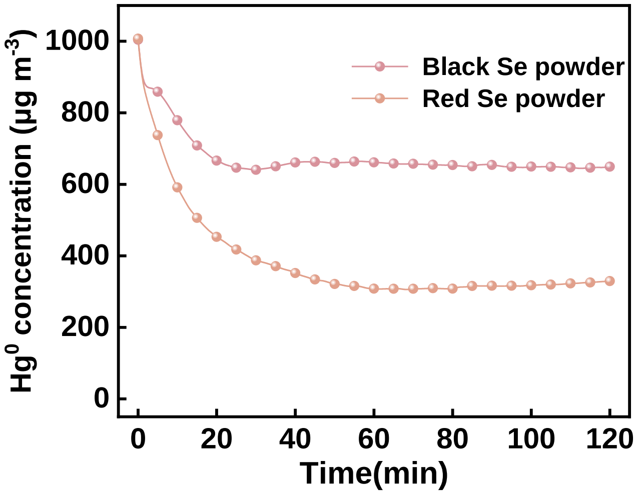

Supplementary Fig. 17. Hg^0 adsorption performances of different samples at varied temperatures.

(a) PPS, (b) Se/PPS-I, (c) Polyester, (d) Se/Polyester.

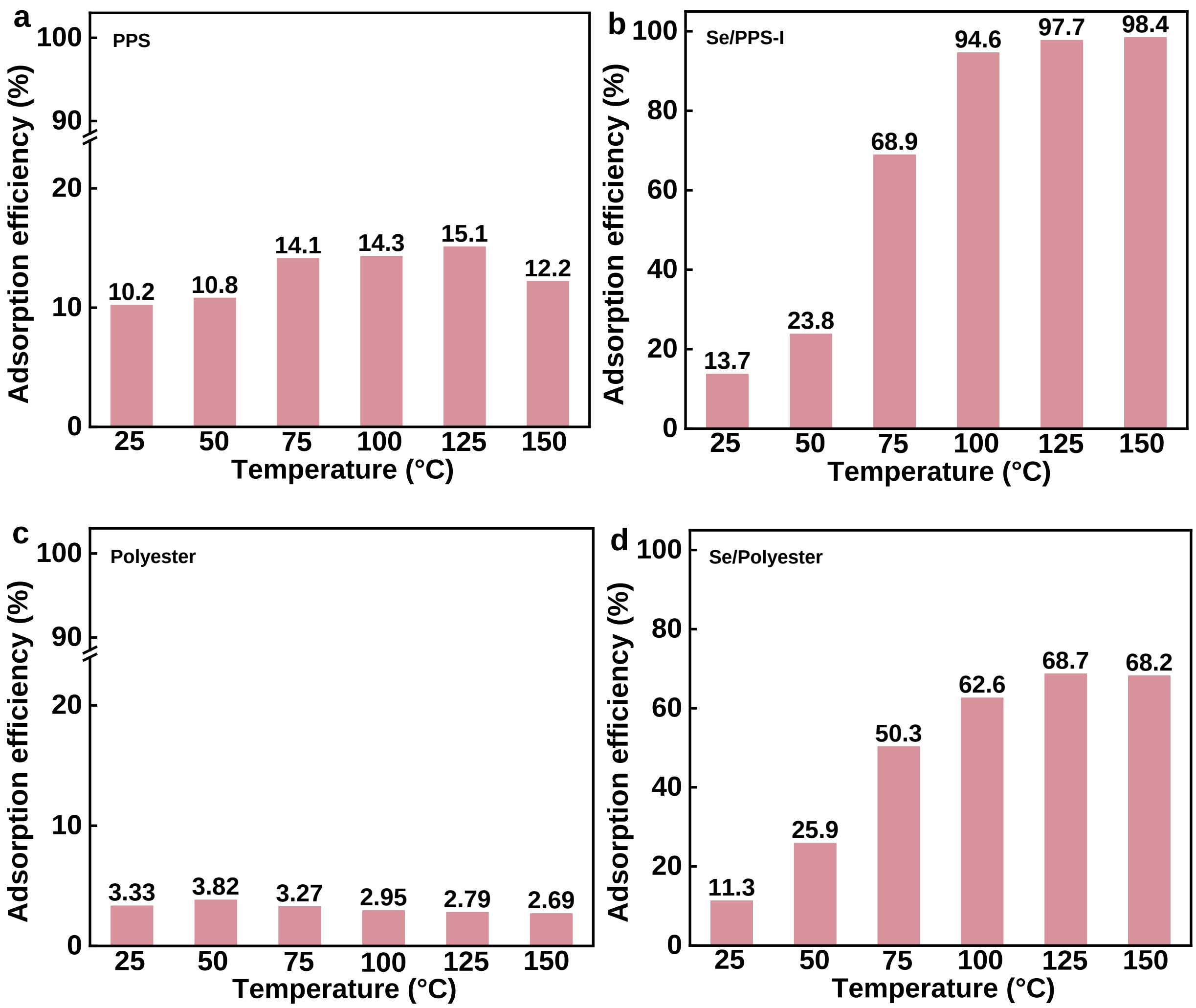

Supplementary Fig. 18. Kinetics models for Hg^0 adsorption over Se/PPS-I. (a) Pseudo-first-order and (b) Pseudo-second-order kinetic models, (c) Intra-particle diffusion model, and (d) Elovich model.

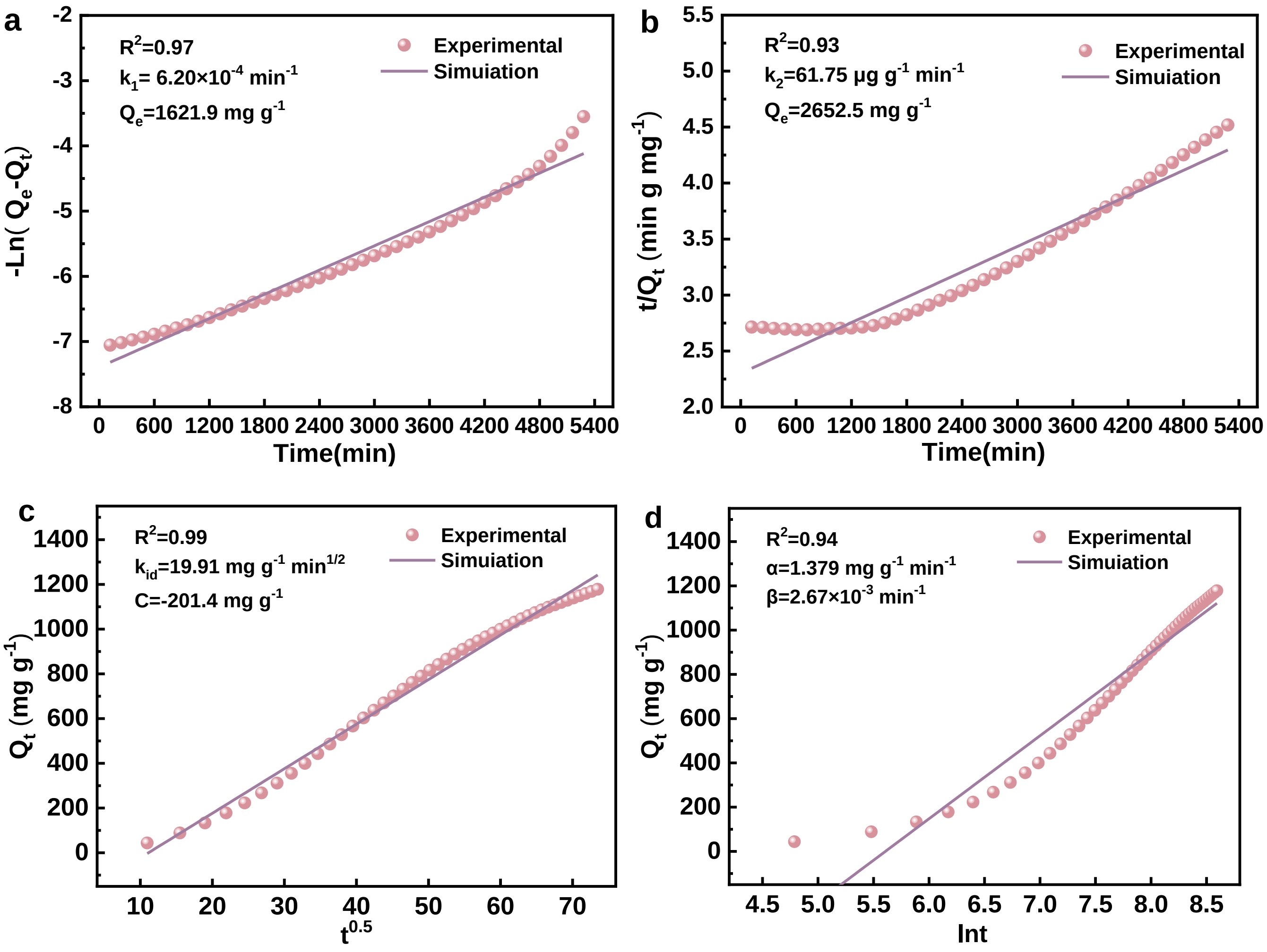

Supplementary Fig. 19. Hg^0 adsorption performance of Se/PPS under different atmospheres (a) different H_2O concentration, (b) different SO_2 concentration, (c) H_2O and SO_2 co-existed.

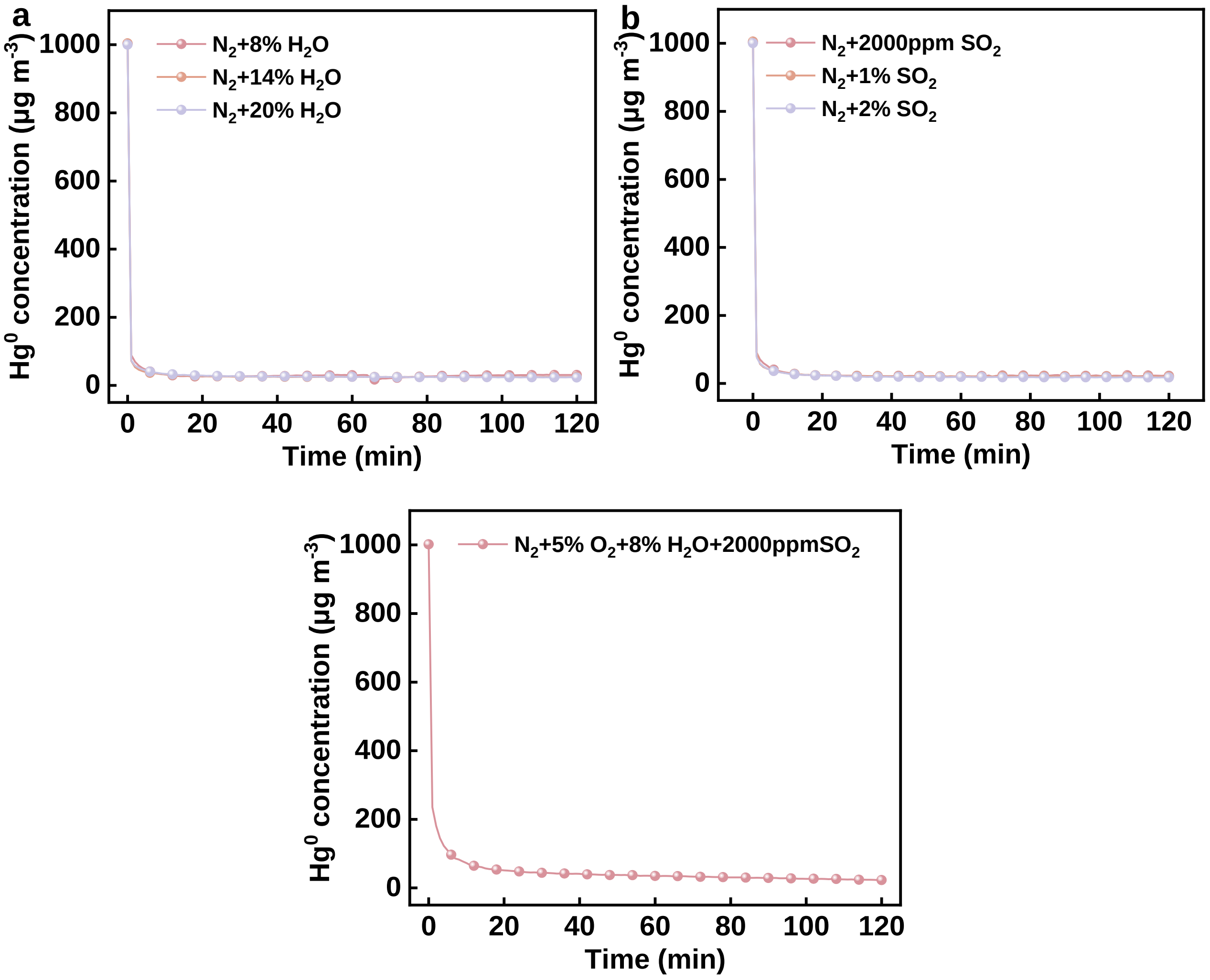

Supplementary Fig. 20. A proposed scheme for simultaneous removal of Hg^0 and dust by Se/PPS.

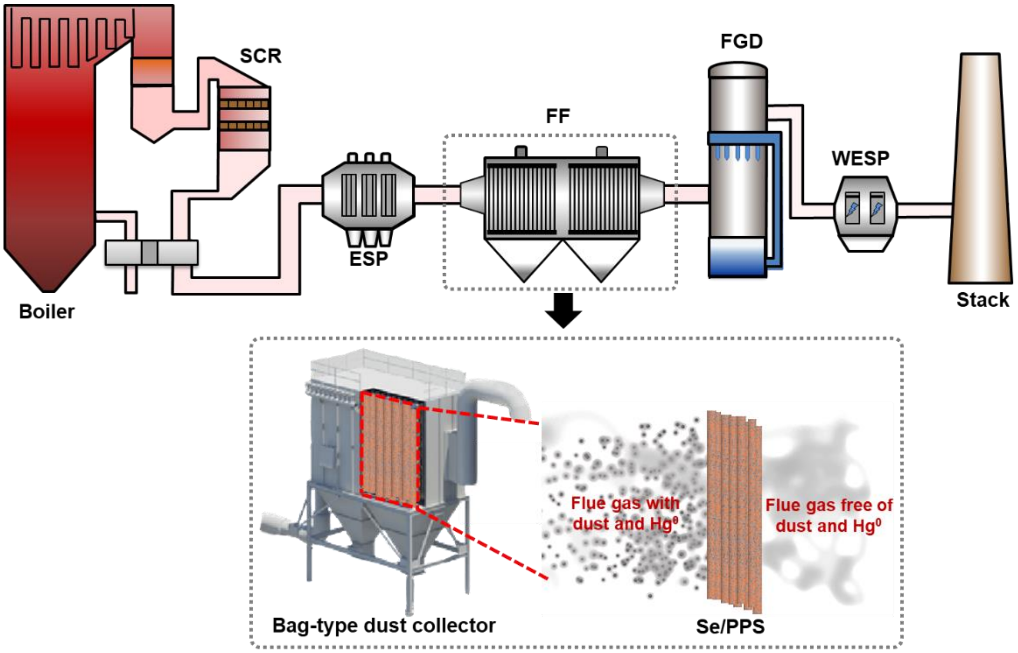

Supplementary Fig. 21. Hg^0 removal performances of Se/PPS with and without covering by particulate matter. (a) Se/PPS covered by fly ash, (b) Hg^0 removal performances of Se/PPS with and without covering by fly ash.

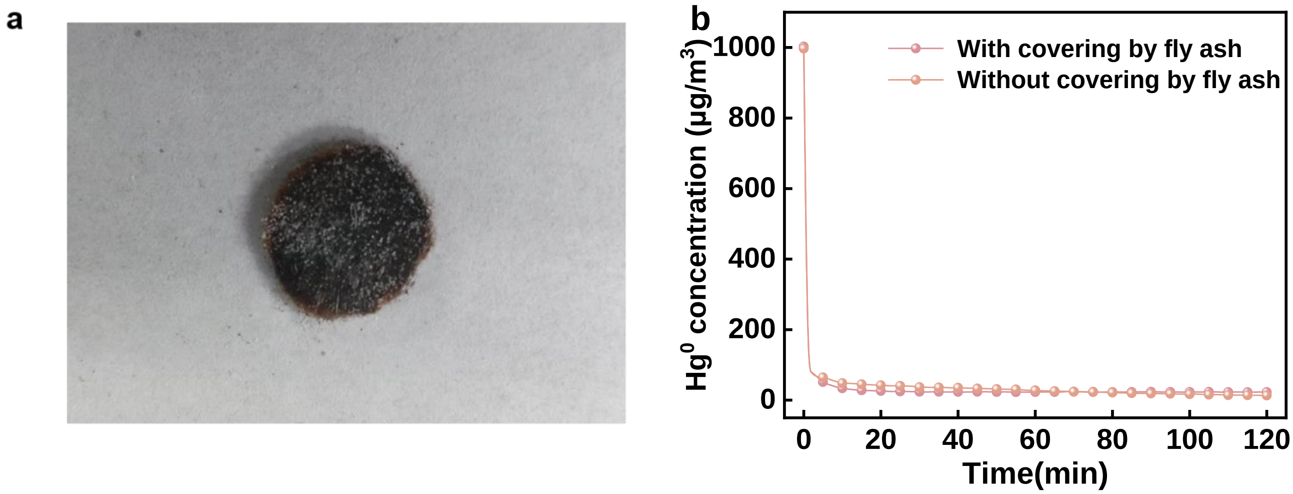

Supplementary Fig. 22. The Hg^0 adsorption capacity of Se/PPS as a function of time.

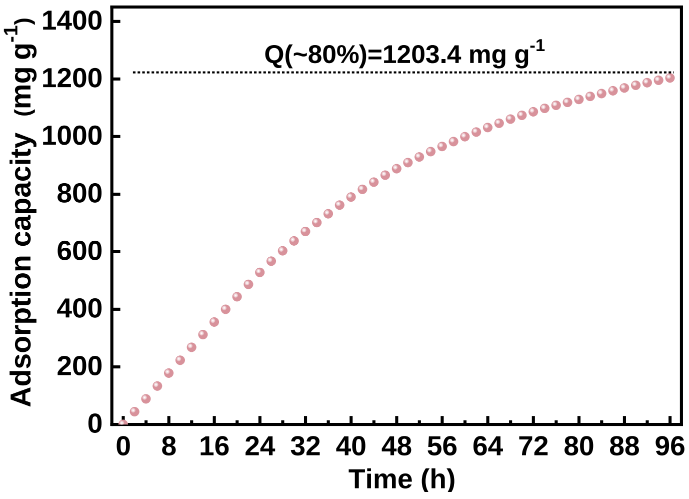

Supplementary Fig. 23. Schematic diagram of mercury and selenium recovery system.

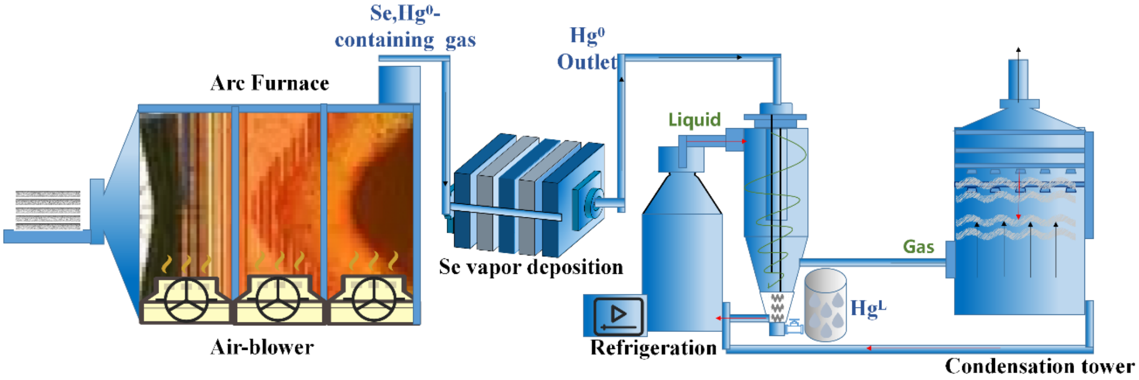

Supplementary Fig. 24. Photo of mercury and selenium recovered from spent Se/PPS-I. (a) liquid mercury, (b) selenium.

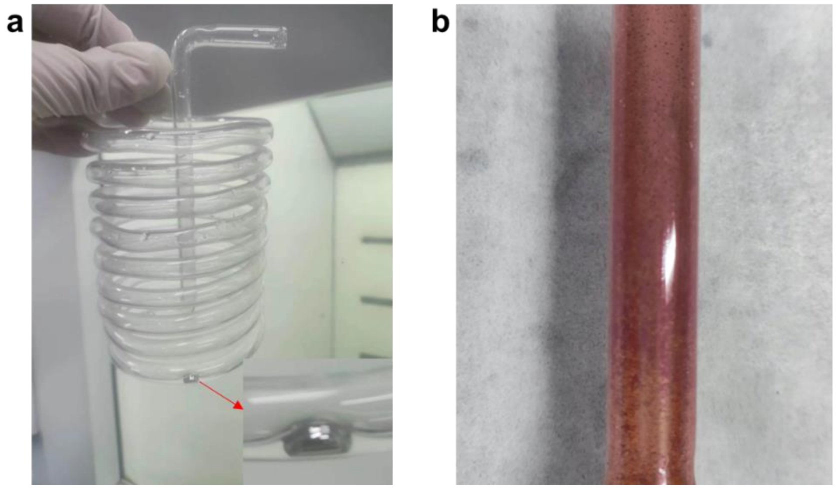

Supplementary Fig. 25. XPS spectra over fresh and Hg-laden Se/polyester. (a) Se 3d, (b) Hg 4f of XPS spectra.

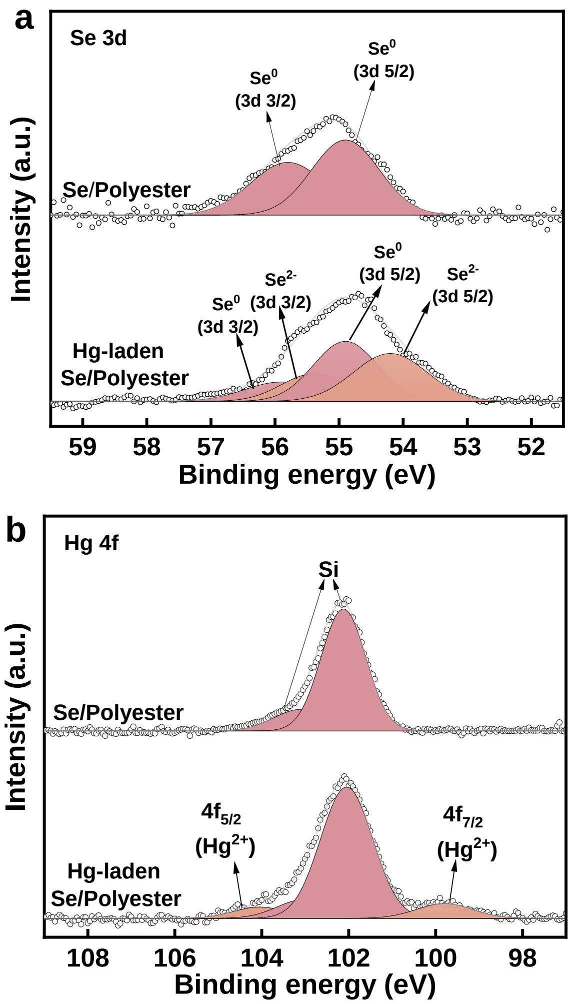

Supplementary References

1. Yang J, *et al.* Charge distribution modulation and morphology controlling of copper selenide for an enhanced elemental mercury adsorption activity in flue gas. *Chem. Eng. J.* **442**, 136145 (2022).
2. Jia T, Gu Y, Wu J, Li F. Copper selenide sensitized low-cost porous coordination polymers towards efficient capture trace gaseous elemental mercury. *Chem. Eng. J.* **457**, 141288 (2023).
3. Yang Z, *et al.* Nanosized Copper Selenide Functionalized Zeolitic Imidazolate Framework-8 (CuSe/ZIF-8) for Efficient Immobilization of Gas-Phase Elemental Mercury. *Adv. Funct. Mater.* **29**, 1807191 (2019).
4. Duan X, *et al.* Efficient immobilization and detoxification of gaseous elemental mercury by nanoflower/rod WSe_2/halloysite composite: Performance and mechanisms. *J. Hazard. Mater.***458**, 131898 (2023).
5. Liu H, Ruan W, Zhang Z, Shen F, Zhou Y, Yang H. Dual 2-dimensional CuSe/g-C3N4 nano-heterostructure for boosting immobilization of elemental mercury in flue gas. *Chem. Eng. J.* **435**, 134696 (2022).
6. Yang J, *et al.* Selenium Functionalized Metal–Organic Framework MIL-101 for Efficient and Permanent Sequestration of Mercury. *Environ. Sci. Technol.* **53**, 2260-2268 (2019).
7. Yang Z, *et al.* Development of selenized magnetite ($Fe_3O_{4-x}Se_y$) as an efficient and recyclable trap for elemental mercury sequestration from coal combustion flue gas. *Chem. Eng. J.* **394**, 125022 (2020).
8. Meng F, *et al.* Cupric ion stabilized iron sulfide as an efficient trap with hydrophobicity for elemental mercury sequestration from flue gas. *Sep. Purif. Technol.* **330**, 125385 (2024).

9. Liao Y, Xia Y, Zou S, Liu P, Liang X, Yang S. In Situ Emergency Disposal of Liquid Mercury Leakage by Fe-Containing Sphalerite: Performance and Reaction Mechanism. *Ind. Eng. Chem. Res.* **56**, 153-160 (2017).

10. Liu W, Xu H, Liao Y, Wang Y, Yan N, Qu Z. Co-doped ZnS with large adsorption capacity for recovering Hg^0 from non-ferrous metal smelting gas as a co-benefit of electrostatic demisters. *Environ. Sci. Pollu. R.* **27**, 20469-20477 (2020).

11. Quan Z, *et al.* Study on the regenerable sulfur-resistant sorbent for mercury removal from nonferrous metal smelting flue gas. *Fuel* **241**, 451-458 (2019).

12. Zhao Y, *et al.* Experimental study on fly ash capture mercury in flue gas. *Science China Technological Sciences* **53**, 976-983 (2010).

13. Hsi H, Tsai C, Lin K. Impact of Surface Functional Groups, Water Vapor, and Flue Gas Components on Mercury Adsorption and Oxidation by Sulfur-Impregnated Activated Carbons. *Energy & Fuels* **28**, 3300-3309 (2014).

14. Zeng H, Jin F, Guo J. Removal of elemental mercury from coal combustion flue gas by chloride-impregnated activated carbon. *Fuel* **83**, 143-146 (2004).

15. Zhou Q, *et al.* Experimental and kinetic studies of gas-phase mercury adsorption by raw and bromine modified activated carbon. *Fuel. Process. Technol.***134**, 325-332 (2015).

16. Xie Y, Yan B, Tian C, Liu Y, Liu Q, Zeng H. Efficient removal of elemental mercury (Hg^0) by SBA-15-Ag adsorbents. *J. Mater. Chem. A* **2**, 17730-17734 (2014).

17. Luo G, *et al.* Carbon Nanotube-Silver Composite for Mercury Capture and Analysis. *Energy & Fuels* **24**, 419-426 (2010).

18. Xu H, *et al.* Regenerable Ag/graphene sorbent for elemental mercury capture at ambient temperature. *Colloids. Surfaces. A* **476**, 83-89 (2015).

19. Hao R, *et al*. Enhanced removal of elemental mercury using MnO_2-modified molecular sieve under microwave irradiation. *Chem. Eng. J.* **450**, 137997 (2022).

20. He J, Reddy G, Thiel S, Smirniotis P, Pinto N. Simultaneous Removal of Elemental Mercury and NO from Flue Gas Using CeO_2 Modified MnO_x/TiO_2 Materials. *Energy & Fuels* **27**, 4832-4839 (2013).

21. Jia T, *et al.* Nanosized $ZnIn_2S_4$ supported on facet-engineered CeO_2 nanorods for efficient gaseous elemental mercury immobilization. *J. Hazard. Mater.***419**, 126436 (2021).
