# Supplementary material for: Biomimetic mercury immobilization by selenium functionalized polyphenylene sulfide fabric
Source: Nat Commun. 2024 Feb 12;15:1292. doi: 10.1038/s41467-024-45486-7 (PMC10861514; doi:10.1038/s41467-024-45486-7)
Supplement: Supplementary file 3 — Description of Additional Supplementary Files [file 41467_2024_45486_MOESM3_ESM.pdf]

Description of Additional Supplementary Files

File Name: Supplementary Data 1
Description: Atomic coordinates of the optimized computational models.
